# Octreotide Protects the Mouse Retina against Ischemic Reperfusion Injury through Regulation of Antioxidation and Activation of NF-***κ***B

**DOI:** 10.1155/2015/970156

**Published:** 2015-06-14

**Authors:** Jun Wang, Ziqiang Sun, Junsheng Shen, Dongdong Wu, Fang Liu, Ruisheng Yang, Shaoping Ji, Ailing Ji, Yanzhang Li

**Affiliations:** ^1^Medical College of Henan University, Kaifeng 475004, China; ^2^Medical College, Henan Polytechnic University, Jiaozuo 454000, China; ^3^The First Affiliated Hospital of Henan University, Kaifeng 475001, China

## Abstract

Somatostatin (SST), an endogenous peptide, may exert anti-inflammatory and neuroprotective effects on retinal injury induced by ischemia. Retinal ischemic reperfusion (I/R) injury always produces many reactive oxygen species (ROS), which can aggravate the tissue damage. The effects of octreotide (OCT), a SST analogue, on retinal I/R injury and ROS formation, are not very clear. In this study, we observed the effects of OCT on morphological changes, oxidative stress, and cell death, induced by retinal I/R injury. The activation of nuclear factor *κ*B (NF-*κ*B) and intercellular adhesion molecule-1 (ICAM-1) were further evaluated in I/R retina treated with or without OCT. The retinal layer thickness was increased at 1 day after I/R and decreased at 7 days after I/R (*P* < 0.05). This effect was associated with increase in MDA and ROS levels (*P* < 0.05). The Tunel-positive cells increased and the number of ganglion cell layer (GCL) neurons decreased significantly after I/R injury. The expression of p-p65 and ICAM-1 increased significantly in I/R retinas (*P* < 0.05). Each effect was markedly attenuated by application of OCT. These data indicate that OCT protects the retina against retinal I/R damage, which could be through inhibition of oxidative stress and downregulation of NF-*κ*B and ICAM-1 expression.

## 1. Introduction

Ischemic injury has been thought of as a kind of final common pathological pathway in many retinal diseases, such as retinopathy of prematurity, diabetic retinopathy, acute glaucoma, and vein occlusion. Neuronal death induced by retinal I/R injury is the major cause of visual impairment and blindness. There are several biological processes that happened during retinal I/R injury, including oxidative stress, calcium overload, inflammation, cellular necrosis, and apoptosis [1]. Studies have shown that SST and its analogs may influence the activities of leukocytic infiltration, adhesion, and chemotaxis and also inhibit the generation of reactive oxygen radicals from leukocytes [2–4]. Octreotide (OCT) is a synthetic SST analog and also is a SST receptor agonist. It has been demonstrated that OCT plays anti-inflammatory and antioxidant roles in many biological processes, such as the treatment of acute pancreatitis, neurogenic plasma extravasation, and I/R injury in intestine [5–7]. In nervous system, studies have indicated that OCT may alleviate the tissue edema induced by I/R injury in the brain and retina [8–10], and the reduction of ROS generation may be involved in this process [8, 9]. In in vitro study, for example, SSTR_2_ agonist reduced the cell lost in rat retinal plant caused by chemical ischemia [11], and studies in knockout animals have shown that SST has neuroprotective effect in ischemia retinas [12]. Most recently, an investigation has indicated that SST eye drops play a neuroprotective role through inhibiting the glial cell activation, glutamate accumulation, and apoptosis in an experimental diabetic model [13]. But it is still unclear whether OCT has neuroprotective effect in retina during I/R injury in vivo. The purpose of this study was to demonstrate whether application of OCT has protective effects on mouse retina during I/R injury in vivo and further determine whether this effect was associated with antioxidant reaction and modulation of the activation of NF-*κ*B and ICAM-1.

## 2. Methods

### 2.1. Animals

C57BL6 mouse, weighted 19–28 g, were purchased from the Model Animal Research Center of Nanjing University and housed in IVC cages, under a 12 h-12 h of light to dark cycle condition. The humidity and temperature were kept at 60 ± 5% and 22 ± 3°C, respectively. Water and food were accessed freely. Protocols about the animal surgery and performance were approved by the Committee of Medical Ethics and Welfare for Experimental Animals, Henan University, School of Medicine (approval number MEWEAHUM 2014-0001). All performance for the surgery must be under anesthesia condition and all efforts should be made to reduce the suffering and number of animals used. Seventy-two mice were used in this study. Animals were divided randomly into three groups: control group (20), ischemia/reperfusion (I/R) group (26), and I/R + OCT group (26). Usually, only the right eyes were chosen to perform I/R procedures. In the control group, sometimes both of the eyes were selected for the analysis.

### 2.2. Mouse Model of Retinal Ischemia-Reperfusion

Mice were anesthetized with pentobarbital (120 mg/kg, i.p.). One drop of 1% tropicamide was used to dilate the pupils. Proparacaine hydrochloride was dropped onto the cornea for topical anesthesia. Animals were lied down on their left side. A 30-gauge sterile needle connected with a sterile plastic infusion tube was put horizontally into the anterior chamber of right eye. A normal saline bag interlinked with the other end of the infusion tube was raised to a height of 150 cm, which made the intraocular pressure (IOP) increase to about 110 mmHg. Ischemia can be estimated by observation of the anterior globe, which was whitened when the vessels were blocked. Ischemic condition was kept for 60 min, and then the needle was removed gradually. The vessels reperfusion could be confirmed by observation of the color restored in anterior segment of globe. In our preliminary experiments, sham surgery was made in the left eyes. For these animals only the needle was put into the anterior chamber, but the IOP was not increased. The results indicated that there were no significant differences in retinal morphology and protein expression between the control group (intact) and sham group (sham surgery). So the sham group alone was not arranged especially and integrated with intact control animals into control group. Animals were killed at 24 hours, 7 days, and other time points after I/R, retinas, or eyeballs were collected for the following analysis.

### 2.3. Drug Administration

For the control group, ischemia-reperfusion was not performed, and no drug was applied. In I/R group, ischemia-reperfusion was induced and then sterile saline was injected subcutaneously as vehicle control. For I/R + OCT group, OCT (dissolved in sterile saline), 30 *μ*g/kg (Novartis Pharma Schweiz AG, Stein, Switzerland), was injected twice/day subcutaneously after completion of the ischemic condition.

### 2.4. Quantification of the Retinal Thickness

To quantify the extent of histopathological damage in I/R retina, the overall retina, inner nuclear layer (INL), outer plexiform layer (OPL), and outer nuclear layer (ONL) were determined. For each eye, three measurements from center, nasal, and temporal of the hemisphere were averaged. Each experiment group was arranged with 3–5 animals.

### 2.5. Evaluation of Neuronal Cell Loss

Retinal neurons in the GCL were evaluated using the confocal microscopy imaging in whole retinal preparations which were labeled with NeuN antibody, the neuron marker. Eyeballs were fixed in 4% paraformaldehyde (PFA) at 4°C for at least one night. Cornea was cut from the eyeball, and then the retina was isolated from the eyecup. After washing with PBS, retina was incubated in 10% normal goat serum in PBS with 1% triton for 30 minutes. Anti-NeuN (1 : 400; Boster) was applied to retina overnight at 4°C. Retina was washed with PBS for 5 min, 3 times. Anti-rabbit IgG conjugated with Cy3 was applied to the retina at room temperature. After one-hour incubation and washing with PBS, retina was flat-mounted. Immunofluorescence images acquisition were taken by a confocal microscope (FV1000, Olympus, Japan). The GCL was confirmed by the trace of inner retinal vessels. Three serial images with 1.5 *μ*m interval focused on the NeuN-positive neurons in GCL were taken. Then, the images were merged to generate a new picture as a representative sample. Each group used 4–6 animals and the cells were counted by using ImageJ software (v2.1, USA).

### 2.6. Tunel Assay

Apoptotic positive cells were detected by using an In situ Cell Apoptosis Detection Kit (Tunel assay, Boster, Wuhan, China) on paraffin-embedded retina sections. Five different areas in each retinal section were chosen to count the Tunel-positive cells. The positive cell numbers per millimeter of retinal surface in the 5 areas were reported. Three or four samples per group were used for this assay.

### 2.7. Determination of MDA Levels and ROS Formation

Retina was dissected completely from each eyeball and then homogenized by using RIPA buffer (Beyotime). Homogenate was centrifugated (1600 g) at 4°C for 10 min, and the supernatant was selected. BCA Protein Assay Kit (Boster, Wuhan, China) was used to determine the protein concentration. The MDA levels were measured using assay kits (Beyotime, Shanghai, China) according to the manufacturers' instructions. Reaction oxygen species (ROS) in the retinal tissue sections were assayed by dihydroethidium (DHE). DHE, as a ROS fluorescent probe, can penetrate through the cellular membrane and be oxidized into ethidium bromide by intracellular ROS. Then the ethidium bromide can be bound to DNA in the nucleus and fluoresce red. Cryosections from frozen retinas were incubated with DHE (5 *μ*M, 40 min, at room temperature). Images were obtained with a fluorescent microscope. The relative fluorescent intensity was quantified by using ImageJ (v2.1, USA) software.

### 2.8. Immunohistochemistry Staining

Paraffin-embedded retinal sections were deparaffinized in xylene “1” and xylene “2” for each 15 min after a roast at 60°C for 60 min. Then sections were rehydrated in ethanol with a decreased concentration. After blocking the tissue sections in 2% goat serum, primary antibody ICAM-1 (1 : 100; Boster) was applied to the sections overnight at 4°C. On the next day, sections were incubated with secondary antibody for 1 hour at room temperature. After washing (twice, 5 min) with PBS, section images were obtained by a microscope, and 3–5 samples per group were used for ICAM-1 staining.

### 2.9. Western Blot Analysis

After isolation of the whole retina from eyeball, RIPA buffer (Beyotime Biotech, China) was applied to retinas for the homogenates (1 hour, on the ice). Protein concentrations were measured using a BSA protein assay kit (Beyotime Biotech). 30 *μ*g protein per sample was separated on 10% SDS-PAGE and then transferred onto nitrocellulose membranes (Millipore). The membranes were blocked in 5% milk or 3% BSA in Tris-buffered saline with 0.5% Tween 20 (TBST) for 1 hour at room temperature on shaking table. The membrane was incubated overnight at 4°C with primary antibodies, including p65 (BIOSS, 1 : 200), p-p65 (BIOSS, 1 : 200), ICAM-1 (Boster, 1 : 400), and *β*-actin (Boster, 1 : 400). On the next day, after washing with TBST, membranes were incubated with horseradish peroxidase-conjugated secondary antibody (1 : 10000) on the shaking table for 1 hour at room temperature. After washing the membranes with TBST, protein bands were detected by using ECL reagent and obtained images after exposure on X-OMAT film (Kodak). The intensity of the protein band was semiquantitatively measured with image analysis software (ImageJ v2.1, USA), and *β*-actin was used for calibration.

### 2.10. Statistical Analyses

All data were expressed as mean ± SD (*n* = 3–6) and differences among groups were statistically analyzed by one-way ANOVA. *P* < 0.05 was considered as statistically significant. Statistical analyses were carried out using Sigma stat32.

## 3. Results

### 3.1. OCT Reduced the Changes of Retinal Thickness Induced by I/R

**Figure 1:**
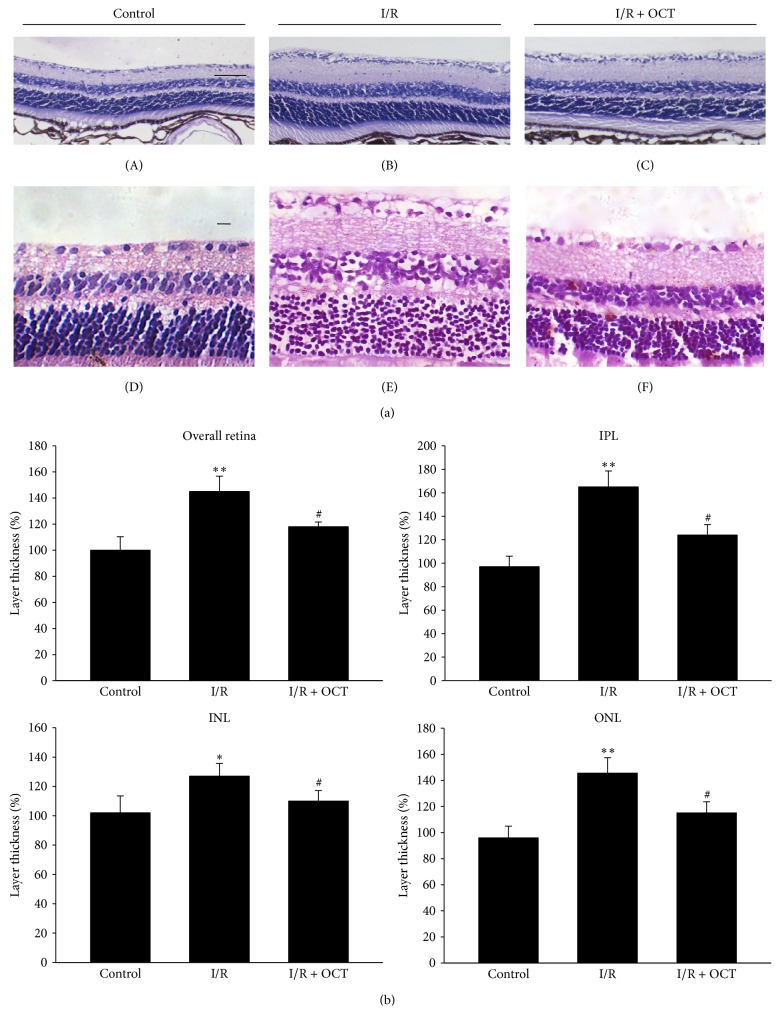
(a) Light micrographs of a cross-section through normal mouse retina ((A) 400x, (D) 200x) and 24 h after I/R without application of OCT ((B) 400x, (E) 200x) or with OCT application ((C) 400x, (F) 200x). Scale bar, 25 *μ*m. (b) Whole retinal thickness or sublayer (ONL, INL, and IPL) thickness at 24 h after I/R. Data represent mean ± standard deviation of percentages relative to the control values. For statistical analysis, one-way ANOVA was applied with Tukey test. ^*∗*^ *P* < 0.05, ^*∗∗*^ *P* < 0.01 compared with control; ^#^ *P* < 0.05 compared with I/R (ONL: outer nuclear layer; INL: inner nuclear layer; IPL: inner plexiform layer).

**Figure 2:**
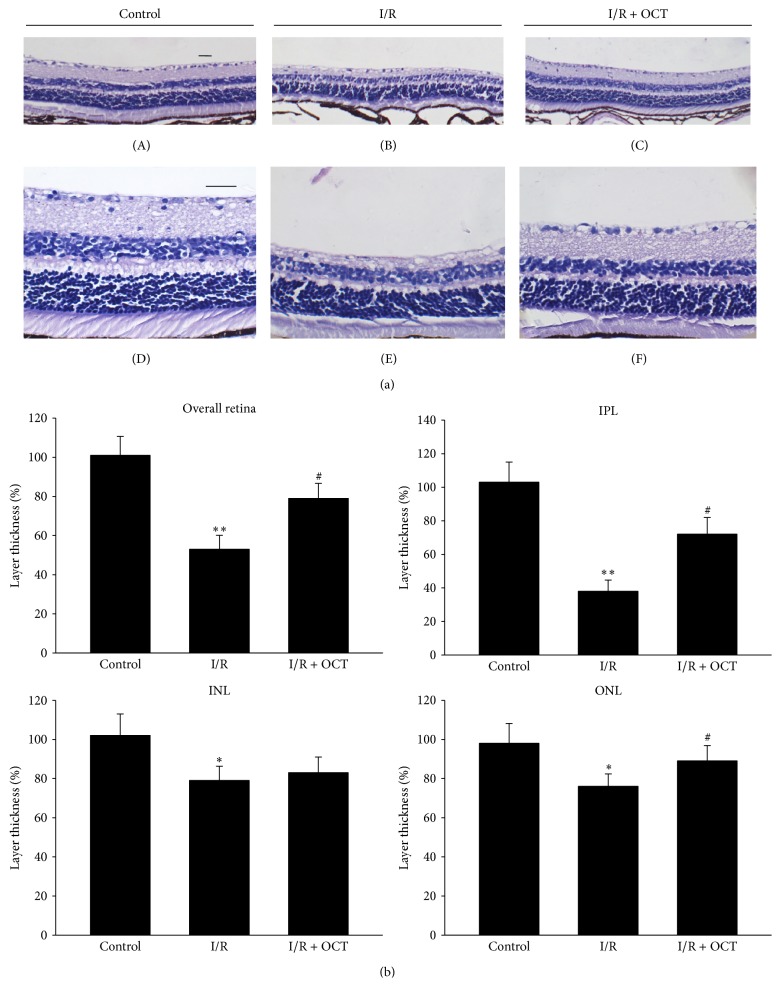
(a) Light micrographs of a cross-section through normal mouse retina ((A) 400x, (D) 200x) and 7 days after I/R without application of OCT ((B) 400x, (E) 200x) or with OCT application ((C) 400x, (F) 200x). Scale bar, 25 *μ*m. (b) Whole retinal thickness or sublayer (ONL, INL, and IPL) thickness at 7 days after I/R. Data represent mean ± standard deviation of percentages relative to the control values. For statistical analysis, one-way ANOVA was applied with Tukey test. ^*∗*^ *P* < 0.05, ^*∗∗*^ *P* < 0.01 compared with control; ^#^ *P* < 0.05 compared with I/R. (ONL: outer nuclear layer; INL: inner nuclear layer; IPL: inner plexiform layer).

The overall retina thickness and the component layers in INL, OPL, and ONL were measured. At 24 hours after retinal I/R injury, as shown in Figures 1 and 2, the thickness of whole retina and sublayers were increased significantly compared with those in the control group. The application of OCT reduced this increasing. At seven days after I/R injury, the overall retinal thickness was significantly lower than those in the control group. OCT treatment eliminated this decrease in retina thickness. The component layers of retina (ONL, IPL, and INL) were coincidently changed with the whole retina thickness after I/R injury without and with OCT application, as shown in Figures 1(b) and 2(b).

### 3.2. OCT Improved Neuronal Survival in Injury Retina

**Figure 3:**
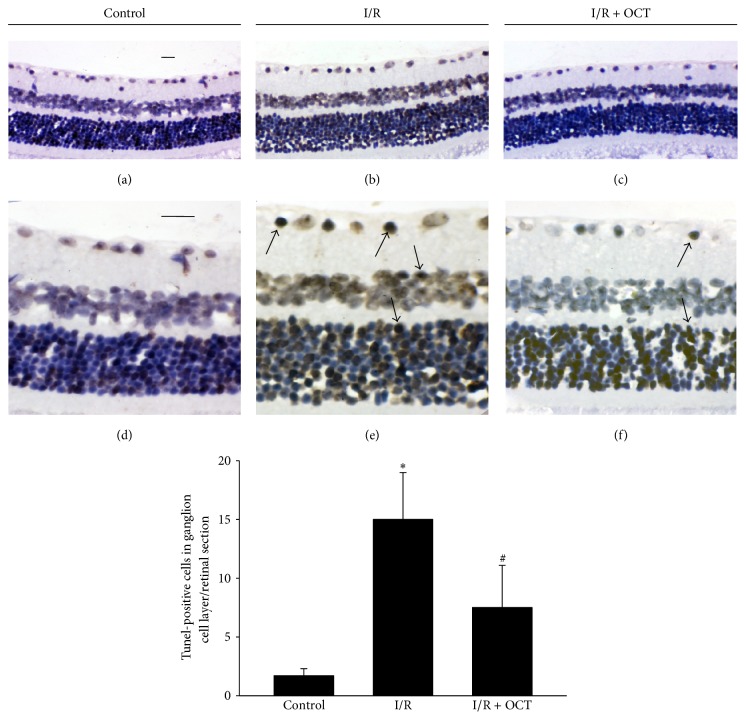
Representative photographs of Tunel staining in control ((a) 200x, (d) 400x), I/R ((b) 200x, (e) 400x), and I/R + OCT ((c) 200x, (f) 400x) group, 24 h after I/R. Arrows: Tunel-positive cells (400x). OCT decreases the number of Tunel-positive cells (^*∗*^ *P* < 0.01 versus control, ^#^ *P* < 0.01 versus I/R). Scale bar, 25 *μ*m.

**Figure 4:**
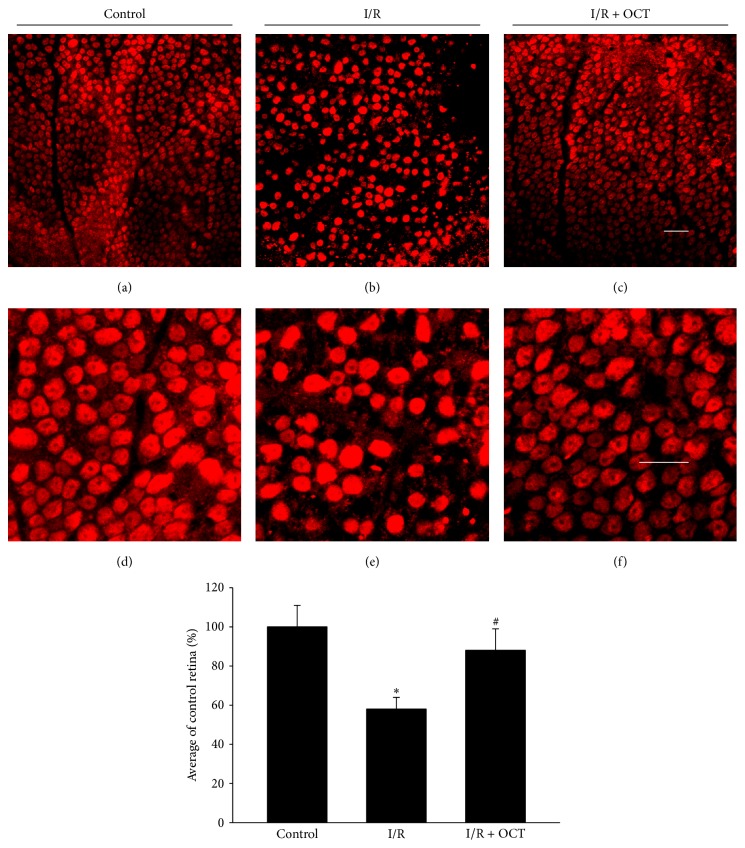
Neuron density in the GCL retina. Confocal imaging of flat-mounted retina labeled with NeuN antibody at 7 days after I/R shows a significantly reduction in density of NeuN-positive cells in the GCL. Application of OCT inhibited the loss of NeuN-positive GCL neurons after I/R ((a)–(c) 200x, (d)–(f) 400x, *n* = 4, ^*∗*^ *P* < 0.05 versus control, ^#^ *P* < 0.05 versus I/R). Scale bar, 50 *μ*m.

The improved retinal morphology by OCT suggested that OCT played an important role during retina I/R injury. Tunel assay was performed to determine the apoptosis cells at 24 hours after I/R injury in different groups. In Figure 3, the number of Tunel-positive cells in I/R group was increased significantly compared with that in control group. There is no obvious difference observed in the quantity of Tunel-positive cells between I/R and I/R + OCT histological images. In I/R + OCT group, compared with that in I/R group, the Tunel-positive cell quantity showed a significant decrease. As seen in Figure 3, Tunel-positive cells were mainly localized in the GCL and INL, while the ONL was less affected. The quantitative results analysis was also shown in Figure 3. Effect of OCT on the GCL neurons loss was determined by confocal image analysis (Figure 4). Flat-mounted retinas were performed and labeled with NeuN antibody to quantify the NeuN-positive cells in GCL at 7 days after I/R. The results indicated that the numbers of NeuN-positive neurons in I/R group were decreased significantly than those in the control group, but the positive neurons in OCT + I/R group were almost normal. The GCL neurons counts analysis was also shown in Figure 4.

### 3.3. OCT Inhibited the Increase of Retinal MDA Level Induced by I/R Injury

**Figure 5:**
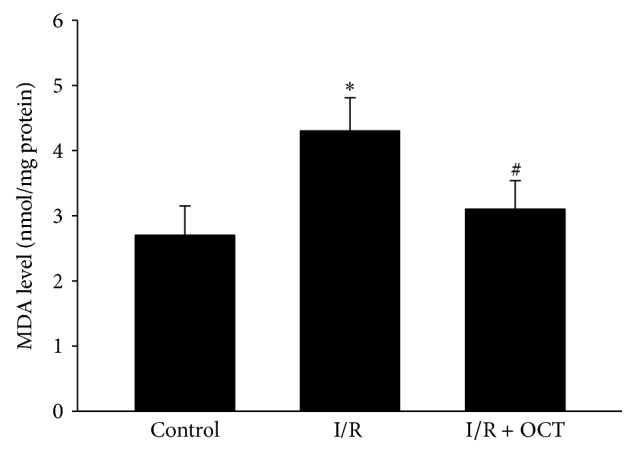
MDA levels of retinal tissue (nmol MDA/mg protein). (^*∗*^ *P* < 0.05 versus control, ^#^ *P* < 0.05 versus I/R, *n* = 4 for each group.)

The mean values of retinal MDA in the control, I/R, and I/R + OCT group were 2.7 ± 0.45, 4.3 ± 0.51, and 3.1 ± 0.44 nmol/mg protein, respectively. The value in I/R group was significantly greater than that in the control group. There was no significant difference between I/R + OCT group and control group. Application of OCT inhibited the increase of MDA level induced by retina I/R injury (Figure 5).

### 3.4. OCT Reduced the Reactive Oxygen Species Formation

**Figure 6:**
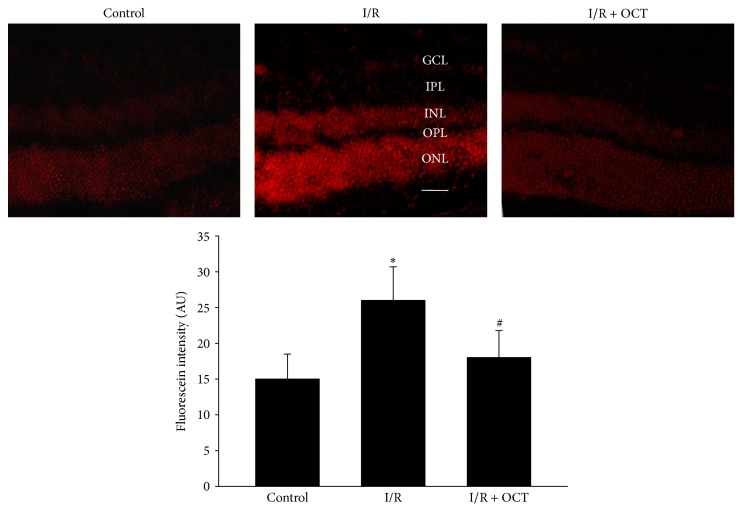
I/R induced-ROS formation was inhibited by application of OCT. DHE imaging of superoxide formation at 24 h after I/R showed increased DHE reaction, which was inhibited by OCT (*n* = 4, ^*∗*^ *P* < 0.05 versus control, ^#^ *P* < 0.05 versus I/R). Scale bar, 50 *μ*m, 200x.

DHE staining was used to test whether ROS were suppressed by application of OCT. DHE, as a ROS fluorescent probe, can be oxidized by intracellular ROS and fluoresce red. At 24 hours after retina I/R injury, DHE fluorescence was significantly upregulated in retinal cryosections. However, application of OCT decreased the levels of ROS.  Figure 6 showed the analysis of relatively fluorescein intensity in the color areas.

### 3.5. OCT Inhibits NF-*κ*B p65 Activation and ICAM-1 Expression

**Figure 7:**
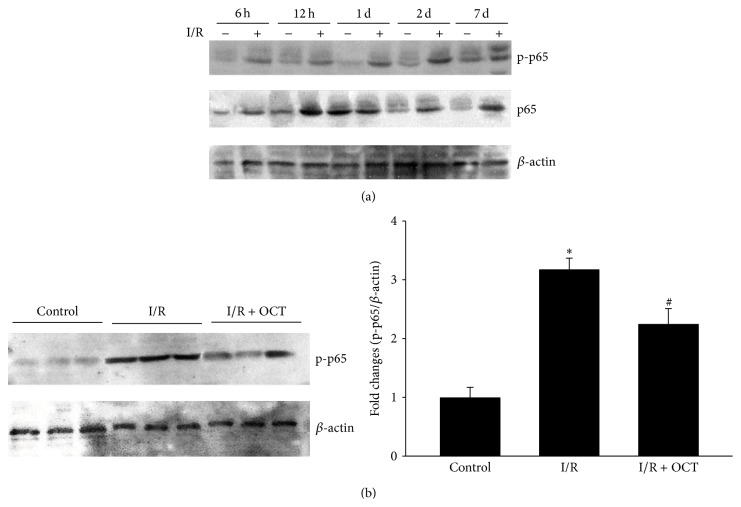
OCT reduces the phosphorylation of NF-*κ*B (p65) during I/R. (a) Western blot analysis shows a time-dependent increase in phosphorylation p65 (p-p65) at 6 h, 12 h, 1 d, 2 d, and 7 d after I/R injury. (b) OCT suppresses p-p65 formation relative to the sham samples (^*∗*^ *P* < 0.05 versus sham, ^#^ *P* < 0.05 versus I/R).

**Figure 8:**
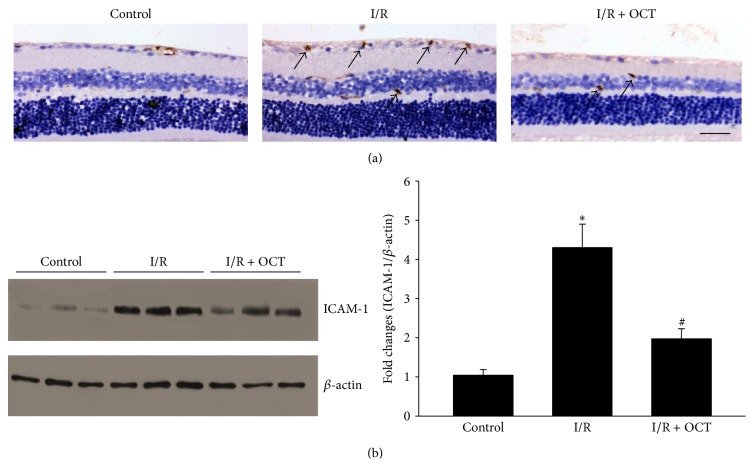
OCT reduces the expression of ICAM-1. (a) Representative pictures of ICAM-1 stained retinal sections 1 day after the I/R injury (400, scale bar 50 *μ*m). (b) OCT suppresses ICAM-1 formation relative to the control (arrows indicate ICAM-1 positive area; ^*∗*^ *P* < 0.05 versus control, ^#^ *P* < 0.05 versus I/R.).

NF-*κ*B is an inflammatory transcription factor, and the activation of its subunits p65 has been demonstrated to play an important role in a lot of tissue damage caused by I/R, which were found in both brain and retina [14–16]. Western blot analysis was performed to evaluate the effect of OCT on p65 activation induced by I/R. The results indicated that the phosphorylation of p65 increased with a time-dependent manner (Figure 7). The increase of p-p65 can be found at 6 hours after I/R and keeps elevating for 7 days. In application of OCT 24 hours after I/R, the increase of p-p65 was significantly suppressed (Figure 7). Several studies have indicated that SST and its analogs could inhibit leukocyte infiltration, adhesion, and generation of free radical [2–4]. Cell adhesion molecules can promote the extravasation of immune cells into the retina, which has been shown to promote the progression of the injury. To further confirm if the OCT can influence the ICAM-1 expression during retinal I/R injury, immunohistochemical staining and western blot analysis were used, and the results indicated that the expression of ICAM-1 was significantly increased in both immunohistochemical staining and immunoblot at 24 hours after I/R injury. Application of OCT markedly reduced the immunoreactivity signals of ICAM-1 (Figure 8).

## 4. Discussion

In this study we examined the neuroprotective effects of OCT in a mouse model with retinal I/R injury. The results showed that retinal I/R injury caused the activation of ICAM-1, p-p65, which was correlated with leucocytes infiltration, tissue edema, ROS formation, cell apoptosis, and cell loss. These effects were inhibited by application of OCT, demonstrating the antioxidant and neuroprotective acts of OCT.

Ischemia is a final common pathomechanism in many retinal diseases. It is also the most important reason, leading to visual impairment and blindness. Deprivation of oxygen and metabolic substrates, disturbance of waste products removal, and inflammation response are the main pathological processes involved in cerebral and retinal ischemia damage. These processes would result in the generation of numerous oxygen radicals, which have been considered as a major mediator in retinal injury induced by ischemia [17, 18]. Therefore, pharmacological targets that point to antioxidative and anti-inflammatory properties are considered to have potential therapeutic effect and may protect against retinal ischemic damage.

Based on the suggestion of previous studies, SST system may play anti-inflammatory and neuroprotective roles in many situations and may have been investigated intensively. There is evidence that SSTR_2_ agonists attenuate the cell apoptosis and cell death induced by chemical ischemia in rat retinal plant [11]. A new in vitro ischemic retina model showed that the cells death was obviously reduced in SSTR_1_ knockout retina, where SSTR_2_ was overexpressed and overfunctional [12]. Accumulating evidence indicates that SSTR_2_ is an important target for the protection of ischemic retina. In particular, it has been established that OCT could reduce the edema and MDA in ischemic rat brain and guinea pig retina [8, 9]. But it is still not very clear whether the OCT has neuroprotective effect in retinal ischemia-reperfusion model in vivo. Although OCT is a water-solute reagent, it is difficult to pass the normal blood retina barrier. But, in injury retina where the blood retina barrier is damaged, we do not know how the OCT works in vivo. This study has demonstrated that application of OCT reduced the retinal edema after one day of I/R and increased the retinal thickness after 7 days of I/R compared with I/R retinas. The Tunel-positive cells decreased after 24 hours of I/R, and the NeuN-positive cells counts in GCL were restored at 7 days after retinal I/R by administration of OCT. It indicates that OCT has neuroprotective effects against retinal I/R injury. However, the exact mechanisms of OCT are not very clear in this condition.

Oxygen free radical generated from tissue was the major cause in I/R injury. In the cell membranes, polyunsaturated fatty acid content was abundant, so the cell membranes were subjected to oxidative attraction and lipid peroxidation. The accumulated peroxidation products may increase the vascular permeability, inducing tissue edema and irritating the inflammatory reaction. These changes could lead to the cell death [8, 19]. The retinal injury caused by I/R in our study corresponded to previous reports [8, 14, 18, 20], in which the thickness of retina increased initially for the cause of edema and then became thinner gradually owing to the damage of the tissues (reduction of the cell number seven days after I/R). To evaluate the superoxide formation, we performed DHE image. Superoxide existed in fresh tissue (frozen sections) or living cells oxidized DHE to form ethidium bromide, which could be bound to DNA in the nucleus and fluoresce red. The results showed that application of OCT inhibited the obvious increase of relative fluorescein intensity in I/R retina, which indicated that the reactive oxygen species (ROS) formation induced by I/R injury was suppressed. MDA is a product of lipid peroxidation and is always used to evaluate the severity of oxidative damage as it is closely relevant to the extent of lipid peroxidation. In the present study, OCT suppressed the increase of MDA values in retinal tissue after I/R injury. This was concordant with previous studies [8, 9].

NF-*κ*B, an important redox-sensitive transcription factor, regulates the genes of many inflammatory mediators and plays an important role in ischemic tissue damage both in brain and in retina [9, 14, 15, 21–23]. In the western blot observation, phosphorylation of NF-*κ*B p65 increased from 6 h and last even to 7 d after I/R injury. Studies have demonstrated that inactivation of NF-*κ*B specifically in astrocytes had neuroprotective effects against I/R induced retinal cell death and other CNS injuries [15, 24–27]. Cybb, Ncf1, and Ncf2 may initiate the activation of NADPH oxidase, which was considered as one of the cellular enzyme systems promoting generation of ROS in I/R [28]. Evidence has proposed that NF-*κ*B correlated the expression of Cybb, Ncf1, and Ncf2 and facilitated the oxidative stress conditions [15]. Another study demonstrated that the membrane catalytic subunit gp91^PHOX^, one of the NADPH oxidase units, played a key role in retinal I/R injury and associated with the phosphorylation of NF-*κ*B p65 [21]. However, the cytosolic subunit p47^PHOX^ did not seem to be influenced by retinal I/R injury [14]. In the present study, activation of NF-*κ*B p65 caused by ischemia was inhibited by application of OCT, which indicated that downregulation of p-p65 by OCT had protective role against the retinal ischemic injury. This is consistent with previous report that OCT may protect the rat brain from acute ischemic injury by inhibiting the NF-*κ*B activation [9].

ICAM-1, a transmembrane glycoprotein belonging to the immune globulin superfamily, is necessary for the adhesion of leucocytes to the capillary endothelium and has been implicated in the development of leukostasis [29]. Immunohistochemistry and western blot analysis showed that the expression of ICAM-1 was obviously increased after retinal ischemia in this study which was compatible with other experiments which indicated that the ICAM-1 mRNA level increased obviously after retinal I/R injury [30]. Many studies have suggested that activation of the transcription factor NF-*κ*B was correlated to an increase expression of inflammatory factor, including ICAM-1, after retinal I/R injury [31–33]. ICAM-1 is an important chemokine in facilitating leukocytes adhesion and migration into tissue interstitial space [34]. As a result of leukocyte infiltration, the capillaries in target tissue may be occluded by the accumulated inflammatory cells, which would exacerbate the ischemic condition. The immune cells activation and migration into the retinal tissue may induce respiratory burst and formation of numerous free radicals, which resulted in severe tissue oxidative damage [35]. Previous reports had demonstrated that blocking functional activation of ICAM-1 or in* Icam1* knockout mice led to the alleviation of cerebral ischemia injury [36, 37]. Our experiment indicated that OCT decreased the expression of ICAM-1 in the retina undergone I/R injury.

In conclusion, the present study provided in vivo evidence that application of OCT protected the retinas in a mouse I/R injury model. Therefore, OCT could be useful as a potentially therapeutic drug for the treatment of ischemic retinopathy. Through downregulating the expression of NF-*κ*B p-p65 and ICAM-1, oxidative stress was obviously inhibited and the neuronal cells obtained protection. During these processes, peripheral leucocytes, vascular endothelia cells, glial cells, and neurons were linked and interacted with each other. Generally, OCT may play an inhibitory role after combining with SST receptors widespread in circulatory and nervous system and exert anti-inflammatory and antioxidative actions. These actions inspire us to further explore the potential therapeutic points about endogenous SST system in the neuroinflammation.
